# The Genetics of Sudden Infant Death Syndrome—Towards a Gene Reference Resource

**DOI:** 10.3390/genes12020216

**Published:** 2021-02-02

**Authors:** Emma B. Johannsen, Linda B. Baughn, Neeraj Sharma, Nicolina Zjacic, Mehdi Pirooznia, Eran Elhaik

**Affiliations:** 1 Department of Biology, Lund University, 22362 Lund, Sweden; emmabjohannsen@gmail.com; 2 Department of Laboratory Medicine and Pathology, Mayo Clinic, Rochester, MN 55905, USA; baughn.linda@mayo.edu (L.B.B.); Sharma.Neeraj@mayo.edu (N.S.); 3 Department of Animal and Plant Sciences, University of Sheffield, Sheffield S10 2TN, UK; nicolina.zjacic@uzh.ch; 4 National Heart, Lung, and Blood Institute, National Institutes of Health, Bethesda, MD 20892, USA; mehdi.pirooznia@nih.gov

**Keywords:** sudden infant death syndrome (SIDS), cot death, annotation, pathway enrichment, network analysis

## Abstract

Sudden infant death syndrome (SIDS) is the unexpected death of an infant under one year of age that remains unexplained after a thorough investigation. Despite SIDS remaining a diagnosis of exclusion with an unexplained etiology, it is widely accepted that SIDS can be caused by environmental and/or biological factors, with multiple underlying candidate genes. However, the lack of biomarkers raises questions as to why genetic studies on SIDS to date are unable to provide a clearer understanding of the disease etiology. We sought to improve the identification of SIDS-associated genes by reviewing the SIDS genetic literature and objectively categorizing and scoring the reported genes based on the strength of evidence (from C1 (high) to C5 (low)). This was followed by analyses of function, associations between genes, the enrichment of gene ontology (GO) terms, and pathways and gender difference in tissue gene expression. We constructed a curated database for SIDS gene candidates consisting of 109 genes, 14 of which received a category 4 (C4) and 95 genes received the lowest category of C5. That none of the genes was classified into the higher categories indicates the low level of supporting evidence. We found that genes of both scoring categories show distinct networks and are highly diverse in function and involved in many GO terms and pathways, in agreement with the perception of SIDS as a heterogeneous syndrome. Genes of both scoring categories are part of the cardiac system, muscle, and ion channels, whereas immune-related functions showed enrichment for C4 genes. A limited association was found with neural development. Overall, inconsistent reports and missing metadata contribute to the ambiguity of genetic studies. Considering those parameters could help improve the identification of at-risk SIDS genes. However, the field is still far from offering a full-pledged genetic test to identify at-risk infants and is still hampered with methodological challenges and misunderstandings of the vulnerabilities of vital biological mechanisms.

## 1. Introduction

Sudden infant death syndrome (SIDS) (9ICD 798.0; 10ICD R95), “crib death”, or “cot death” was first described in 1953 by Werne and Garrow as the “sudden apparently unexplained death during infancy” [1,2,3]. Adelson and Kinney [4] added in 1956 that these deaths occurred in “a child who was thought to be in good health or whose terminal illness appeared to be so mild that the possibility of a fatal outcome was not anticipated”. In 1970, Beckwith [5] included the requirement of an autopsy, revising SIDS to be “the sudden death of any infant or young child which is unexpected by history and in which a thorough postmortem examination fails to demonstrate an adequate cause of death”. This definition became widely accepted, though new definitions were proposed to include the age of the infant and the environmental conditions [6]. By 2004, SIDS was defined as: “The sudden unexpected death of an infant under 1 year of age, with onset of the fatal episode apparently occurring during sleep, that remains unexplained after a thorough investigation, including performance of a complete autopsy and review of the circumstances of death and the clinical history” (but without genomic analyses) [7]. There is evidence of SIDS dating back at least 2000 years. It was mentioned in the Hebrew Bible (Kings 3:19) and has been a focus of medical studies since the late 20th century [8]. Nonetheless, its etiology remains unexplained, and it remains a diagnosis of exclusion.

Following public health initiatives in the 80s and 90s to reduce the incidence of SIDS, like the Safe to Sleep campaign that recommended putting infants to sleep in a supine position to reduce choking or suffocation, the incidence of SIDS has been dramatically reduced by 50–90% in the Western world [9,10,11]. Despite the success of these measures, SIDS remains a major cause of infant deaths in developed countries. In the US, ill-defined and unspecified causes of mortality (R95-R99 ICD10 code) accounted for 2075 deaths in 2016 (0.07% of all live births) [12]. In 2017, SIDS was the fourth leading cause of infant deaths in the US, accounting for 6% or 35.5 infant deaths per 100,000 live births [13].

It is widely accepted that SIDS can be caused by environmental and/or biological factors. The triple risk hypothesis proposes that SIDS occurs when three risk factors (a vulnerable infant, a critical developmental period of the first months of life, and environmental stressors) overlap [14]. For example, infants with intrinsic vulnerabilities in their critical developmental period that are exposed to physical stress (e.g., abuse, trauma, smoking) have an increased risk of SIDS [8]. This theory, however, cannot explain the four main characteristics of SIDS—male predominance (60:40) [15], the 39% lower SIDS rate among US Hispanic compared to non-Hispanic people [12], the seasonal variation with most cases occurring in winter [16], and that 50% of cases occur between 7.6 and 17.6 weeks after birth with only 10% past 24.7 weeks. These characteristics are explained by the allostatic load hypothesis for SIDS [17,18], which purports that prolonged and repetitive exposure to stressors in the perinatal, prenatal, and postnatal environments (e.g., neonatal circumcision, poor postnatal weight gain [19], hyperthermia [20], epilepsy [21], and maternal smoking [17,18]) is maladaptive and has a cumulative effect that increases the risk of SIDS. Neither theory identifies SIDS-associated or -causative genes.

Multiple genetic studies aimed at understanding the genetic background of SIDS have yielded a large number of candidate genes. These genes have been grouped into four major pathways [23,24]:

### 1.1. Cardiac Defects

Cardiac defects include cardiomyopathies and cardiac channelopathies. The first can be split into dilated cardiomyopathies (*DCM*), hypertrophic cardiomyopathies (HCM), and arrhythmogenic right ventricle cardiomyopathies (ARVC) [25,26]. The latter is caused by variants in genes encoding ion channels or associated proteins and contains long QT syndrome (LQTS) for which the genes *KCNQ1*, *KCNH2*, *SCN5A*, *CAV3*, *GPD1-L*, *SNTA1*, *SCN1B*, *SCN2B*, *SCN3B*, and *SCN4B* are of great importance (mutations in the first three genes explain 80–90% of LQTS with the remaining genes having rare mutations [27]). LQTS-induced-SIDS, the most common cardiac channelopathy, has an average estimated prevalence of 12% (ranging from 3.9 to 20.6%) [28]. Other conditions include Brugada syndrome, where over 350 pathogenic mutations were found in the *SCN5A* (18–30%) [29] and a dozen other genes (though 65% of cases do not have a genetic origin) [30], and catecholaminergic polymorphic ventricular tachycardia (CPVT), affected by *RyR2* [31,32]. Furthermore, rare channelopathies affected by *TRPM4*, *KCNJ2*, *CACNA1*C, *GJA1*, and *NOS1AP* may also be of importance [33,34]. Up to 10% of SIDS cases may be due to cardiac defects [35].

### 1.2. Central Nervous System (CNS) and CNS Pathway Defects

The CNS pathways control cardiovascular homeostasis, respiratory control, and sleep [36]. If such pathways are defective, they may impair the arousal reflexes and lead to SIDS. Serotonin (5-hydroxytryptamine/5-HT) is a neurotransmitter regulating brainstem autonomic functions. Genes regulating serotonin pathways include *TPH2*, *SLC6A4*, and *HTR1A* [25,37]. A variable tandem repeat polymorphism of the serotonin transporter, 5-HTT, has also been observed [25,38]. Other noteworthy genes are *PHOX2B*, controlling differentiation of neuronal progenitor cells, *PACAP* and *PAC1*, affecting respiratory control, and *MAOA*, *PHOX2B*, and *SLC6A4,* involved in thermoregulation [39,40,41,42,43,44,45,46,47].

### 1.3. Immune System Dysfunction

The role of immune dysfunction has been hypothesized when infections were observed in upwards of 50% of SIDS cases [48]. Genetic polymorphisms that modulate gene expression have been found in genes encoding proteins involved in immune functions such as the cytokines *IL-10*, *IL-6*, *TNF-*α, and *INF-*γ, as well as a positive correlation between SIDS and *IL-1*α, *IL-1* receptor antagonist genes, *VEGF*, and associated genes [37,49,50,51,52,53,54,55].

### 1.4. Metabolism Deficiencies and Other Disorders

Inborn errors of metabolism account for 1–2% of early childhood deaths (less than three years of age) [56], down ~5% from the ‘80s [57], likely due to improved early diagnosis and treatment. These include fatty acid oxidation disorders, of which medium-chain acyl-CoA dehydrogenase (MCAD) deficiency is the most common [58,59]. Furthermore, they can be associated with mitochondrial defects [35]. Nicotine has been established as a risk factor for SIDS. A common polymorphism in the nicotine metabolizing enzymes gene *FMO3* that results in an amino acid change that was over-represented in SIDS cases of heavy smoking mothers underlines the potential interaction between genetic susceptibility and an environmental hazard in SIDS [60]. Moreover, postnatal nicotine exposure reduced the immunoreactivity of the serotoninergic (5-HT) receptors 1A and 2A in the brainstem, a common risk factor for SIDS [61].

Past reviews, however, neglected to assess the quality of these studies, their sample sizes, and replicability between studies and populations. None of these genes is considered causal, and a search for “Sudden Infant Death Syndrome” on ClinVar [62] returns a single risk gene (SCN5A) whose pathogenicity is debatable. Therefore, although the emerging image of SIDS as a complex multifactorial syndrome with multiple underlying candidate genes may be correct, the lack of biomarkers identified by past studies raises questions as to why SIDS genetic studies are unable to provide a clearer understanding of the disease etiology.

One of the main aims of SIDS research is to develop a molecular test that will allow newborn screening and identification of SIDS-prone infants that can be monitored and/or treated for the medical deficiencies that they exhibit. However, candidate genes must first be identified. Inspired by similar endeavors, like AutDB, which reviewed the genetic autism literature and categorized and scored autism-associated candidate genes [63,64], we sought to evaluate the quality of SIDS-associated genes by reviewing the SIDS genetic literature and to objectively categorize and score the reported genes. We constructed SIDS-DB, a curated publicly available database for SIDS gene candidates. We further analyzed the functions of the candidate genetic variants, the associations between genes, the enrichment of gene ontology (GO) terms and pathways, and the differential gene expressions in key tissues between the genders.

## 2. Materials and Methods

### 2.1. Collection of Data and Scoring

**Figure 1:**
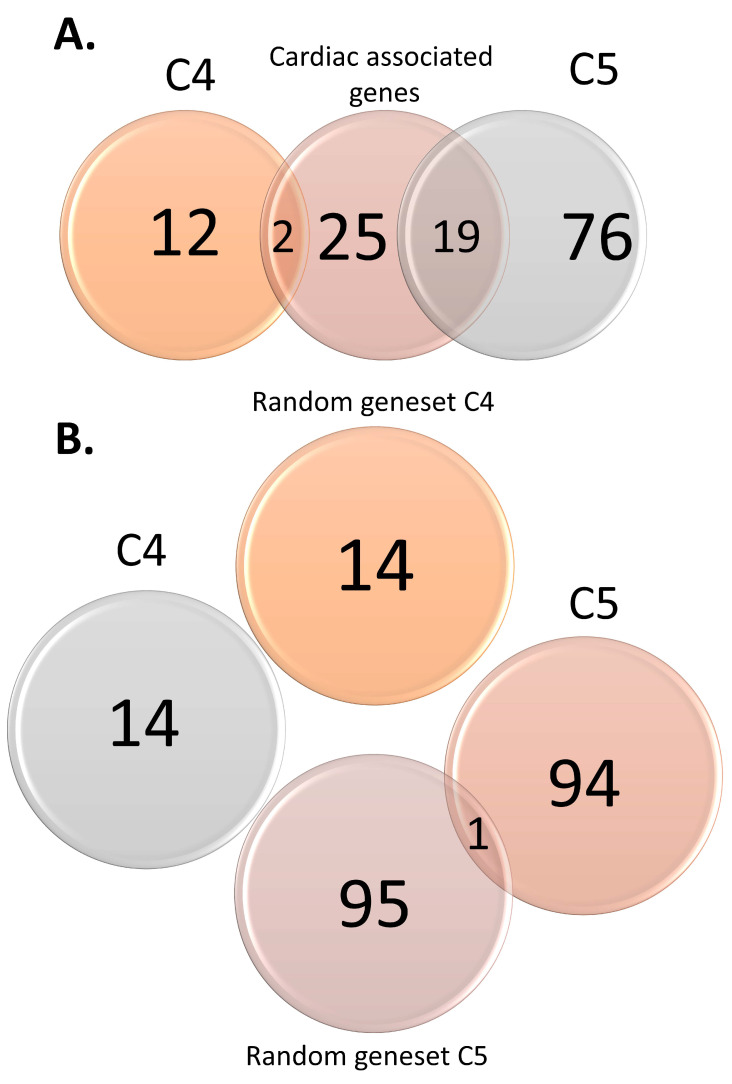
Venn diagrams displaying overlaps between sudden infant death syndrome (SIDS) candidate genes and cardiac-associated variants (**A**) and random gene sets (**B**).

SIDS candidate genes and variants were curated from the literature of the past 30 years (1989–2020) by searching the terms “SIDS” or “Sudden Infant Death Syndrome” combined with “gene” in PubMed ((SIDS AND Gene) OR (“Sudden Infant Death Syndrome” AND Gene)) [65]. The search yielded 752 and 729 studies, respectively. After a careful screening of the abstracts and reading in detail the most promising studies, we identified 95 studies that reported genetic association with SIDS. We curated 109 genes and 253 variants from those studies and annotated and scored them (Tables S1 and S2) according to the criteria that we defined (Table S2). Last, we categorized them according to the strength of the evidence (from C1 (high) to C5 (low)). As positive controls, we curated two lists consisting of genes and variants associated with severe cardiac traits and functions [66,67,68,69]. As negative controls, we created four lists of random genes and variants from the Human Genome Diversity Project (HGDP) [70] (Table 1). All lists had sizes corresponding to those of scoring categories four (C4) and five (C5). Venn diagrams (Figure 1) were calculated using the R package “VennDiagram” [71]. Unless stated otherwise, the tools mentioned in the following analyses were applied to all lists.

### 2.2. Functional Analysis of Genomic Variants

Genomic variants were annotated using the Ensembl Variant Effect Predictor (VEP) [72] (Tables S1). We used the R package “ggplot2” [73] to visualize the gene and variant annotation.

### 2.3. GO and Pathway Enrichment Analyses

We applied four gene set enrichment analysis tools: Enrichr, DAVID, WebGestalt, and GeneMANIA (the association network “Pathway”) to the SIDS candidate and control genes.

For these enrichment analyses, the databases gene ontology (GO) Biological Process, Cellular Component, and Molecular Function were used as well as Kyoto Encyclopaedia of Genes and Genomes (KEGG) and the Reactome Pathway Knowledgebase for pathways [74,75,76]. An adjusted *p*-value < 0.05 was required for significance. Results were plotted using the “ggplot2” package in R for a maximum of the top ten significantly enriched GO terms or pathways per tool and database.

### 2.4. Network Analysis

Network analysis was performed using the multiple association network integration algorithm (GeneMANIA) [77] to test if interactions between candidate genes show explanatory relationships and functions. We analyzed the “co-expression”, “physical interaction”, “genetic interaction”, “shared protein domain”, and “co-localization” networks (Tables S3.1–S3.3).

### 2.5. Tissue Gene Expression

Tissue-specific (blood, brain, and heart) gene expression data from the Genome Tissue Expression Project https://www.gtexportal.org/ (GTEx Analysis Release V8 (dbGaP Accession phs000424.v8.p2)) were obtained from lncRNAKB resources [78]. Transcripts per million (TPM)-feature counts were generated after excluding samples with <10^6^ reads assigned to genes. From the 109 candidate genes/regions, 80 were selected for differential expression analysis between male and female subjects. Genes that had more than 20% of the subjects with a zero-expression value, as well as mitochondrial genes and genes within the 6p22 gene region, were also excluded from the analysis.

A significant difference for tissue-specific gene expression between the male and female subject groups was evaluated using the two-sample Wilcoxon rank-sum test using JMP software (Cary, NC, USA). The significance was estimated at 0.05.

## 3. Results

### 3.1. Criteria for Scoring Categories

SIDS-associated genes curated from the literature were split into six scoring criteria (Table S2) that reflect the level of evidence for the association. The criteria include adherence to the definition of SIDS (according to the San Diego definition of 2004) [7], the number of cases studied, whether an autopsy was performed according to the International SIDS Autopsy Protocol, an evaluation of the statistical analysis, and an evaluation of the matching between cases and controls (based on ancestry, age, and health), as well as basis of evidence and replication. Each gene received a single combined score depending on the fulfillment of these criteria.

We identified 109 SIDS-associated genes and classified them into categories according to the criteria above, resulting in 14 and 95 genes for the lowest categories (C4 and C5, respectively) (Tables S1 and S4.1–S4.3). In other words, the literature search did not reveal any high confidence genes. There was almost no overlap between these categories and the control genes (Figure 1).

### 3.2. Functional Analysis of Genomic Variants

Remarkably, the C4 and C5 variants (Figure S1B,C, Table S4) exhibited a similar functional distribution to the cardiac-associated variants. In both SNP sets, missense was the dominant function for variants (33–43%, compared with 1% in the random set). As expected, most of the random variants (Figure S1C) were intronic. Overall, a far larger proportion of the C4 and C5 variants, as well as cardiac-associated variants, were coding (~34 and ~48%, respectively) compared to the random variants (~2%).

### 3.3. GO and Pathway Enrichment Analyses

**Figure 2:**
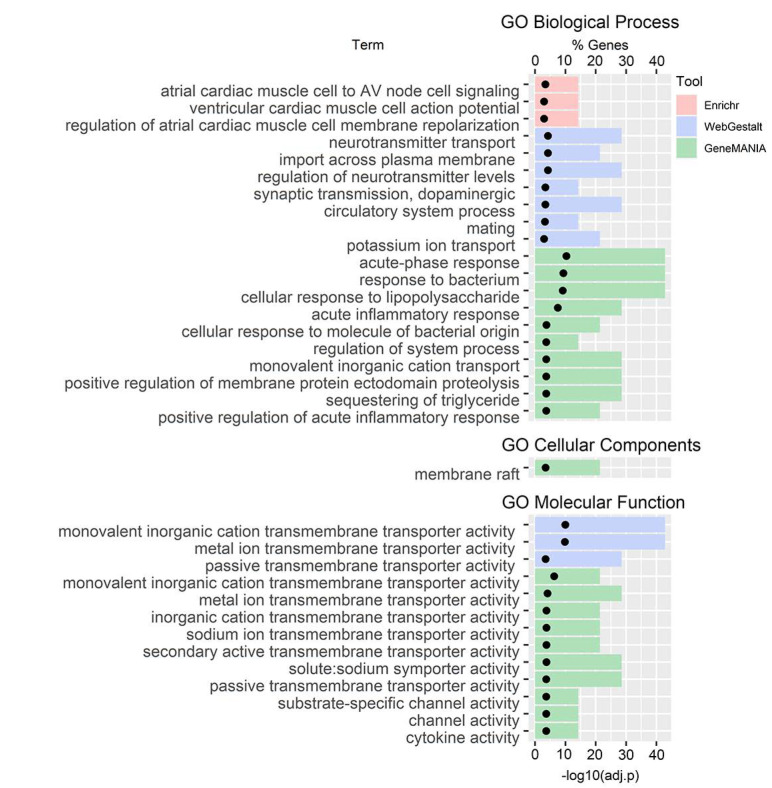
Gene ontology (GO) terms gene set enrichment analysis for C4 genes.

No pathway enrichment was observed for the C4 genes (Figure 2); however, we observed enrichment in several GO terms: In GO Biological Processes, there was an enrichment in cardiac muscle (Enrichr), neurotransmitters, and ion transport (WebGestalt), and immune functions and ion transport (GeneMANIA); in GO Cellular Component, there was an enrichment in membrane raft (GeneMANIA); and in GO Molecular Function, there was an enrichment in transmembrane transporter activity (WebGestalt and GeneMANIA) and channel and cytokine activity (GeneMANIA).

**Figure 3:**
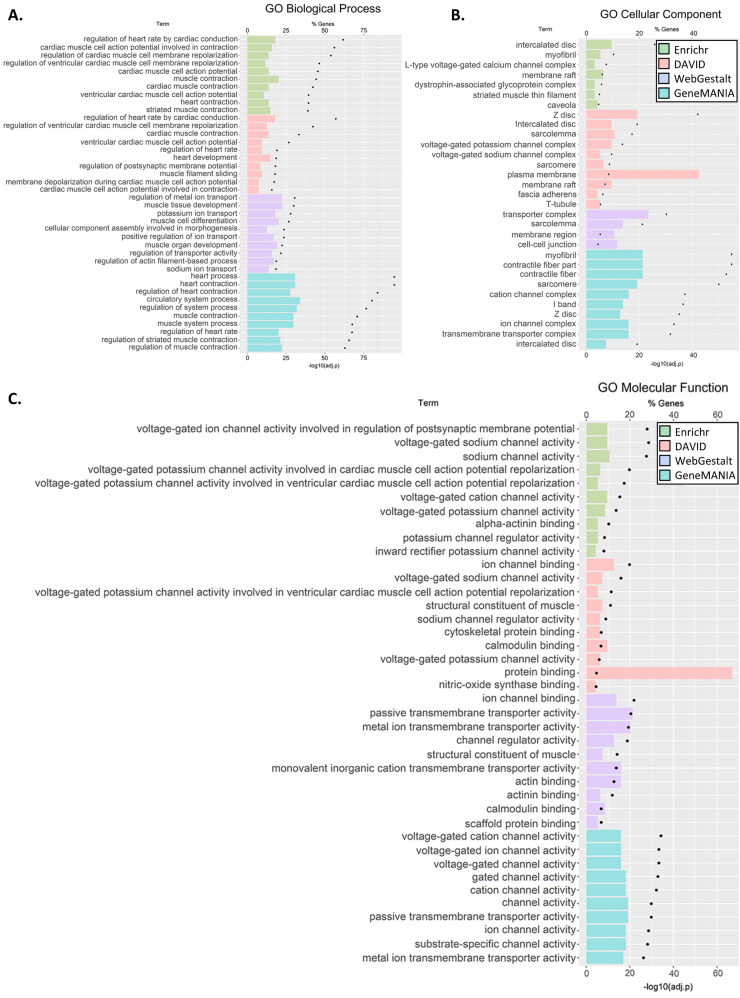

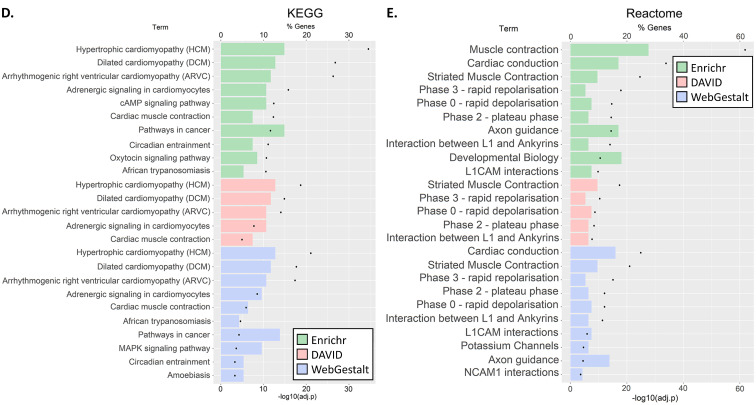
GO terms (**A**–**C**) and pathway (**D**,**E**) gene set enrichment analysis for C5 genes. The analyses were carried out using four tools: David, Enrichr, GeneMANIA, and WebGestalt. Each tool identified pathways that belonged to different GO categories or Kyoto Encyclopaedia of Genes and Genomes (KEGG) or Reactome pathways. We clustered the results by pathway class. Note, GeneMANIA outcomes were annotated and classified to only three GO classes. We then plotted the ten most significant pathways within each GO class (for full results, see Table S5.2.6).

For the C5 genes, at least ten significantly enriched terms were reported by all the tools in GO Biological Process and GO Cellular Component (Enrichr, WebGestalt in KEGG and Reactome) as well as in the GO Cellular Component (DAVID and GeneMANIA) (Figure 3A–C). Testing for pathway enrichment (Figure 3D,E), enrichment of hypertrophic cardiomyopathy (HCM), dilated cardiomyopathy (DCM), arrhythmogenic right ventricle cardiomyopathy (ARVC), adrenergic signaling in cardiomyocytes, and cardiac muscle contraction was unanimously reported using the KEGG database. Enrichment of striated muscle contraction, cardiac action potential, and interaction between L1 and ankyrins was also unanimously reported using Reactome. Further enrichments were identified in Cardiac conduction, axon guidance, and L1CAM interactions (Enricher and WebGestalt) and contraction (Enrichr). Overall, the GO term enrichment was found in the majority of biological processes include cardiac conduction, muscle, heart, and ion transport; the majority of cellular components was of muscle, ion channels, and transporters; and the majority of molecular functions was in ion channel activity, transmembrane transporter activity, and binding of muscle components. We noted that although the annotation tools differed in their reporting, there was significant agreement between them. All the results are shown in Tables S5.1.1.1–S5.5.6.

### 3.4. Network Analysis

**Figure 4:**
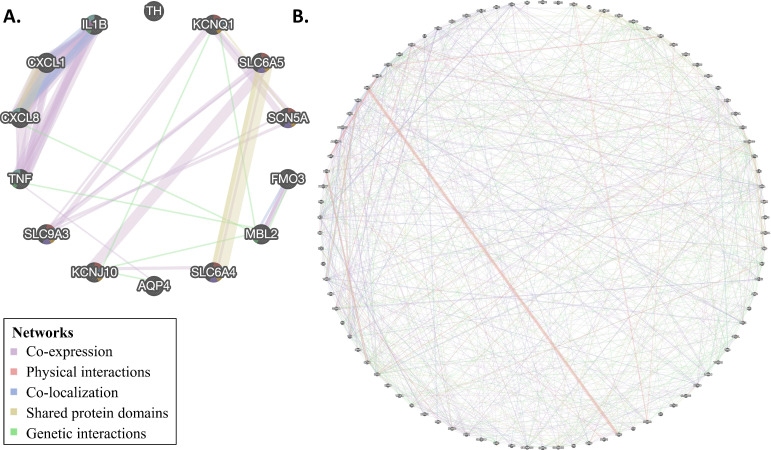
Network analysis was performed on genes in C4 (**A**) and C5 (**B**) genes. Colors correspond to the network types.

The network analysis depicts the relations between genes in a network based on weighted interaction networks from different data sources. Such analysis is useful for decoding genetic patterns and contexts because the network analysis shows how closely two genes are connected, within and across pathways, and can find more subtle signals than in enrichment analysis (Figure 4 and Figures S2 and S3).

For C4 genes, we observed interactions between all genes except for tyrosine hydroxylase, *TH*. We observed co-expression between two groups of genes (*KCNQ1*, *SLC9A3*, *SCN5A*, *SLC6A4*, *KCNJ10*, *SLC6A4* and *IL8*, *TNF*, *IL1A*, *IL1B*); however, shared protein domains (*IL1A*, *IL1B*, and *SLC6A4*, *SLCA5,* and *KCNQ1*, *SCN5A*), co-localization (*IL8* and *IL1B*) and genetic interactions (*KCNQ1*, *MBL2*, *IL8*, *TNF*, *KCNJ10*, *AQP4*, *FMO3*) were also present. This is in contrast to the random C4 gene set, for which only 8/14 genes showed interactions, and no larger groups of interactions were observed (Table S6.2).

For C5 genes, we observed interactions between all genes, except for the mitochondrial genes *MT-ND1* and *MT-CO3*, in complex interplays. As before, the most common type of interaction was co-expression; however, physical interactions, co-localization, shared protein domains, and genetic interactions were also observed. Once again, this is in contrast to the random C5 gene set, which showed markedly fewer interactions (Table S6.3).

### 3.5. Tissue Expression Analysis

**Figure 5:**
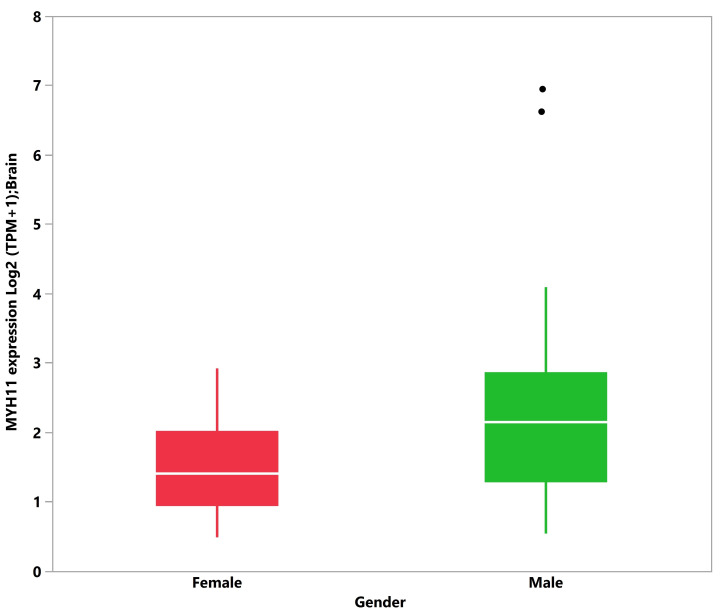
Boxplot and log2 (transcripts per million (TPM)+1) transformed median MYH11 transcript levels in brain tissue (Genome Tissue Expression Project), male (*n* = 67, median = 2.136 TPM) and female (*n* = 19, median = 1.409 TPM). Non-log transformed medians are 3.395 and 1.656 TPM in males and females, respectively. Dots indicate outlier values.

Given the predominance of SIDS among male subjects, we evaluated whether any of the 109 C4 and C5 candidate genes exhibit differential expression between males and females. We obtained gene expression data (TPM-FeatureCounts) from blood, brain, and heart tissues from the GTEx dataset from 20- and 40-year-old male and female subjects. A significant median difference in TPM was observed between male and female subjects for 12 genes: *CACNA2D1* (0.0015 males vs. 0.0027 females, 1.75 fold, *p*-value = 0.0304), *DSG2* (0.0155 males vs. 0.0273 female, 1.76 fold, *p*-value = 0.0425), and *RYR2* (0.0028 vs. 0.0039, 1.39 fold, *p*-value = 0.0319) in blood (109 subjects); *F5* (0.3411 males vs. 0.4653 females, 1.36 fold, *p*-value = 0.0239) and *MYH11* (3.3955 males vs. 1.6560 females, 2.05 fold, *p*-value = 0.0167) in brain (86 subjects); and *BAG3* (100.6781 males vs. 81.0674 females, 1.24 fold, *p*-value = 0.0066), *CBL* (25.1876 males vs. 20.2861 females, 1.24 fold, *p*-value = 0.0385), *HSPD1* (126.1459 males vs. 98.4831 females, 1.28 fold, *p*-value = 0.0069), *IL1RN* (0.3682 males vs. 0.2664 females, 1.38 fold, *p*-value = 0.0399), *IL6* (5.9281 males vs. 3.3274 females, 1.78 fold, *p*-value = 0.0427), *NOTCH1* (15.6559 males vs. 13.9087 females, 1.12 fold, *p*-value = 0.0313), and *VEGFA* (148.9535 males vs. 115.7865 females, 1.29 fold, *p*-value = 0.0253) in heart (65 subjects). Eight genes showed elevated expression in males compared to females: *IL6*, *VEGFA*, *HSPD1*, *BAG3*, *CBL*, *NOTCH1*, *IL1RN,* and *MYH11*. *MYH11* showed the greatest fold change between males and females in brain tissues (Figure 5). Four genes showed elevated expression in females compared to males: *CACNA2D1*, *RYR2, DSG2,* and *F5. DSG2* showed the greatest fold change between males and females in blood. However, when adjusting for false discovery rate (FDR), these differences were not considered significant. Overall, of all the candidate genes considered, differential gene expression between males and females was observed in blood, brain, and heart tissues, but the differences were small and insignificant after FDR correction. In addition, it is unclear how such differences contribute to an increased risk of SIDS among males.

The GTEx data used for this analysis had two limitations: The relatively small sample size of the tissues and the use of relatively older individuals (20–40 years old), representing the younger population for which GTEx data are available but not the target SIDS population. Moreover, this analysis represents individuals who never experienced SIDS, and it is unclear to what extent they represent SIDS victims. Additional studies on the target population and on affected and unaffected infants are necessary to confirm these observations and identify other significant candidate genes that contribute to SIDS.

## 4. Discussion

Sudden infant death syndrome (SIDS) is a complex, multifactorial syndrome used as a diagnosis of exclusion. Despite continuous research and global Safe to Sleep campaigns, SIDS remains one of the most common and poorly understood diagnoses of death among infants between birth and one year of age [79,80] and its etiology remains unexplained. To improve our understanding of the underlying genetic mechanisms of SIDS and to enable the identification of at-risk biomarkers, we performed a comprehensive assessment and downstream functional analysis of genes reported in the literature to be associated with SIDS. We scored these candidate genes based on the quality of the literary evidence and analyzed their function, enrichment along biological pathways, their annotations and interactions, and gender differential gene expression. The goal of this work is to develop predictive gene panels that can identify infants that are genetically at elevated risk to succumb to SIDS.

Most (87%) of the 109 genes identified in the literature were classified to the lowest category C5, suggesting that the studies lacked vigorousness in all scoring criteria (Table S2). The remaining genes (13%) were categorized as C4, suggesting that the genetic association is based on limited and insufficient evidence. C4 genes are highly diverse in function (Table S1) and are involved in many pathways, in agreement with the perception of SIDS as a heterogeneous syndrome. That none of the genes were classified into the higher categories (C1–3) indicates the low level of supporting evidence available in the literature. For instance, no dedicated whole-genome or -exome studies on tissues of SIDS patients or their families were ever done. The most extensive study examined 108 genes, whereas the smallest one examined one gene. By comparison, AUT-DB [63], which uses similar scoring criteria for genes associated with autism, classified 12% of the genes as syndromic, 19.6% as high confidence, 20.4% as strong candidates, and 48% as possible genes at the time of writing. Overall, the SIDS literature is of markedly low quality compared to the autism spectrum disorder literature, which raises major concerns about its validity and usefulness and the standing of research in the field. This criticism joins previous criticism in the literature about the lack of progress of SIDS research and the chase after unsupported paradigms [81]. Future studies should adopt a more comprehensive survey scheme, a genomic analysis, and a more robust statistical approach.

In addition, studies typically do not provide useful metadata on environmental risk factors (e.g., risky parental behavior [82], circumcision status, socioeconomic factors, income, education, and healthcare access) that can stratify the results. Moreover, over the years, the classification of unexplained death became more pluralistic [83] and included terms like “undetermined” and “unknown”, often as semantic alternatives to SIDS. A study examining the definitions of SIDS in papers published in 2005 concluded that 58% of the studies did not specify the definition of SIDS or used a different definition from the 2004 San Diego definition [7]. The use of different definitions hampers comparisons and contributes to inconsistencies in the literature. It is noteworthy that the Third International Congress on SIDS acknowledged these problems and provided revised definitions and exclusion criteria to reduce the ambiguity in death classifications [84]. Other common insufficiencies emanate from a lack of controls or adequately matching them with cases. Concerningly, even if the cause of death is theoretically known but its first manifestation results in death (like in epilepsy or other seizure-related disorders), the infant’s death would be considered SIDS [85]. The SIDS diagnosis in such cases is due to our misunderstanding of seizure-related disorders rather than a genuinely unknown factor. A recent study that examined the genes encoding the voltage-gated sodium channels in a cohort of 73 SIDS victims illustrates this point [86]. The study reported 11 predicted to be pathogenic or likely pathogenic in *SCN1A, SCN1B*, *SCN3A*, *SCN4A*, *SCN9A*, and *SCN10A*. These variants were identified in children without prior histories of epilepsy or unprovoked seizures. Only one child had a history of atypical febrile seizures with hippocampal abnormalities and without history or family history of cardiac arrhythmia. Weese–Mayer et al. [25] already noted that genetic studies of SIDS are limited in terms of numbers of cases, numbers of candidate genes, ancestry identification, and application of statistical methods. Overall, inconsistent reports and missing metadata contribute to the ambiguity of genetic studies and their low validity for a condition that is already complicated by definition, considering those parameters could help improve the identification of at-risk SIDS genes.

Notwithstanding those limitations, our analyses yielded several important insights, which may shed some light on SIDS. First, we found distinct networks connecting SIDS-implicated genes (Figure 3) and significant enrichment in GO terms and pathways associated with cardiac and immune system dysfunctions.

Second, for the C5 genes, we observed enrichment of several pathways and GO terms involved in cardiac defects (e.g., *HCM*, *DCM*, and *ARVC*), cardiac function, and muscle and ion channels and transport (Figure 3). Third, SIDS gene networks consist of multiple cardiac and ion channel genes (Figure 4). Specifically, we found enrichment of C4 genes associated with cardiac muscle, ion transporter, and channel and transmembrane transporter activity. Interestingly, immune-related genes were particularly enriched with C4 genes in both enrichment (Figure 2) and network (Figure 4) analyses. However, C4 gene analyses were limited due to the small number of genes and the limited knowledge of the candidate genes.

Third, there is some support for the association of SIDS genes with neural development. Some of the C4 genes (e.g., *SLC6A4* and *SLCA5*) are associated with defects in the CNS and their pathways. We also observed enrichment in GO terms like “neurotransmitter transport,” “regulation of neurotransmitter levels,” “synaptic transmission, dopaminergic,” and “axon guidance” (Figure 4) for those genes; however, further gene expression evidence is needed to support this hypothesis. No evidence was found to support the metabolism deficiencies and disorders hypothesis.

Finally, analyzing gender differential gene expression patterns in people as young as 20 years old provided no evidence of gene expression differences in tissues relevant to SIDS, with the exception of MYH11 (Figure 5). Mutations in this gene have been associated with human intestinal cancer [87], acute myeloid leukemia [88], and acute aortic dissections (TAAD) [89]. While the absence of evidence is not evidence of absence, mainly provided the limitations of this analysis, it is noteworthy that no genetic finding or biological mechanism has been identified that explains the gender bias in SIDS.

By contrast, Elhaik [18] showed that gender bias varies widely between population groups and states and that male neonatal circumcision (MNC) explains 16% of the variability in male SIDS deaths in the US. This finding supported the “wear and tear” hypothesis for SIDS [17], which explains the main characteristics of SIDS, namely male predominance (60:40) by MNC, the significantly different SIDS rate among USA Hispanics (80% lower) compared to whites by the different circumcision practices of these populations, the 50% of cases occurring between 7.6 and 17.6 weeks after birth, with only 10% after 24.7 weeks by the weaning from the maternally acquired antibodies that protect from infections, and the seasonal variation with most cases occurring during winter by the rise of infections (see the “Infection Hypothesis” [90]) that increase the allostatic load [17] (summarized in Table 1 [17]).

In summary, the road for a genetic test for postnatal at-risk SIDS-genes is still long and hampered with the absence of reliable findings, methodological challenges, and ignorance of the vulnerabilities of vital biological mechanisms. Indeed, Van Norstrand et al. argue that even with newfound genetic information, it is difficult to decide which of the implicated pathways and which combinations of variants are responsible for a SIDS-causing milieu and thus useful for prediction. The authors suggested establishing a postmortem genotyping of SIDS victims as part of the standard autopsy protocol [35]. This recommendation did not fall on deaf ears. In 2013, the National Association of Medical Examiners published a position paper recommended to include sudden unexpected death for postmortem genetic testing [91]. Paludan–Müller et al.’s (2019) reappraisal of previously reported SIDS-associated cardiac ion channel variants found that about two-thirds of these variants are found in population-based cohorts. No significant association with severe cardiac traits was found, indicating that the variants are not highly penetrant and monogenic causes of SIDS [67]. Consequently, we uphold Van Norstrand et al.’s proposal to routinely genotype SIDS victims and propose the inclusion of an environmental survey of known and potential risk factors while raising awareness of the clinical symptoms that may appear before SIDS [92]. We are aware of the difficulty of carrying out genetic testing on deceased patients since US insurance companies may not cover such procedures. We hope for a long-overdue policy change in that area. Whole-genome and proteomic analyses on the infant and their parents may identify novel autosomal dominant, recessive, or de novo lethal mutations, which can pave the way for in utero gene editing, perhaps using CRISPR/Cas9 (e.g., [93]). However, until concrete evidence for genetic markers associated with sudden death in infants is produced, efforts should focus on minimizing or eliminating the known environmental risk factors.

Our study had two more limitations. First, the scheme focused on studies that used the term SIDS in their metadata and abstract. Studies that used terms like “sudden unexplained death” or “recurrent cardiac arrest” to denote SIDS (e.g., [94]) were missed. Second, since the absence of evidence was not evidence of absence, the existence of genetic risk factors for SIDS could not be ruled out. The heterogeneity of the SIDS cases and the inability to distinguish between genetic and environmental risk factors was bound to reduce the power of genetic studies in the field, which already suffers from small sample sizes. We propose to prioritize genetic research in families that have suffered from multiple SIDS cases.

## Figures and Tables

**Table 1:** The dataset size of the positive and negative controls of the SIDS candidate genes and variants.

Control Type	List	Size
Positive	Cardiac-associated genes	46
	Cardiac-associated variants	122
Negative	Random gene set C4	14
	Random SNP set C4	65
	Random gene set C5	96
	Random SNP set C5	188

## Data Availability

The SIDS-DB dataset and all necessary scripts to replicate our figures are publicly available via GitHub https://github.com/eelhaik/SIDS-DB.

## Supplementary Materials

Supplementary Materials are available at https://www.mdpi.com/2073-4425/12/2/216/s1. Figure S1. VEP annotation for SIDS-associated variants. The variant functions for 253 SIDS-associated variants (combined C4 and C5 variants) (**A**), 133 cardiac-associated variants (**B**), and 273 random variants (**C**). Left: All variant functions. Right: Coding variant functions. Figure S2. Network analysis performed on cardiac-associated genes. The colors correspond to the network types. Figure S3. Network analysis was performed on random C4 (**A**) and C5 (**B**) genes. The colors correspond to the network types. Table S1. Variants, consequences, and scores for genes associated with SIDS. Table S2. Gene scoring criteria. Table S3. Network analysis. Table S3.1. Cardiac-associated genes. Table S3.2. Random gene set C4. Table S3.3. Random gene set C5. Table S4. Variant effect predictor, VEP. Table S4.1. SIDS candidate variants. Table S4.2. Cardiac associated genes. Table S4.3. Random SNPs. Table S5. Enrichment analysis.
