# Supplementary material for: The Genetics of Sudden Infant Death Syndrome—Towards a Gene Reference Resource
Source: Genes (Basel). 2021 Feb 2;12(2):216. doi: 10.3390/genes12020216 (PMC7913088; doi:10.3390/genes12020216)

**Figure S1**

The variant functions for 253 SIDS-associated variants (combined C4 and C5 variants) (A), 133 cardiac-associated variants (B) and 273 random variants (C). Left: All variant functions. Right: Coding variant functions.

**
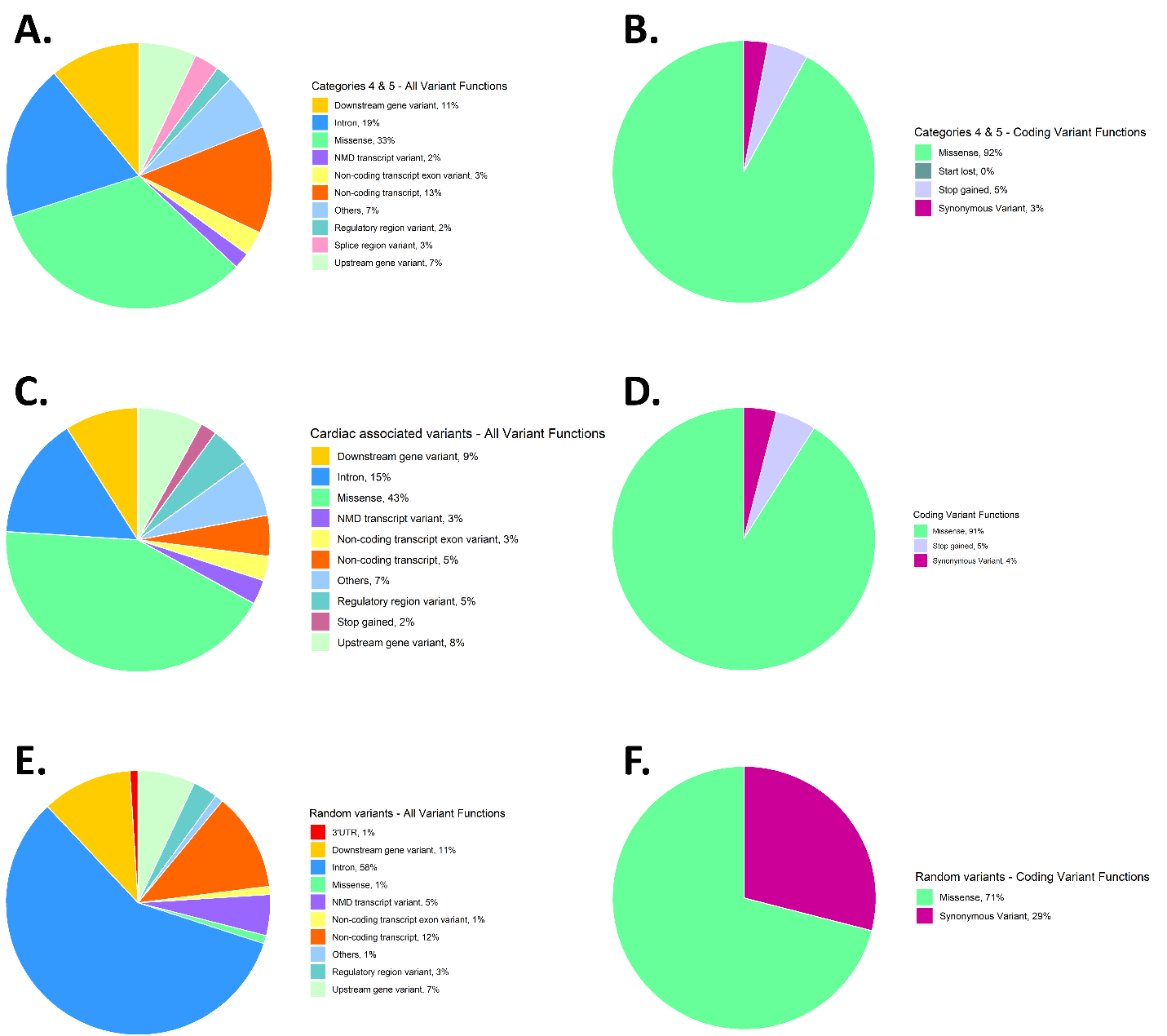
**

**Figure S2**

Network analysis performed on cardiac-associated genes. The colors correspond to the network types.


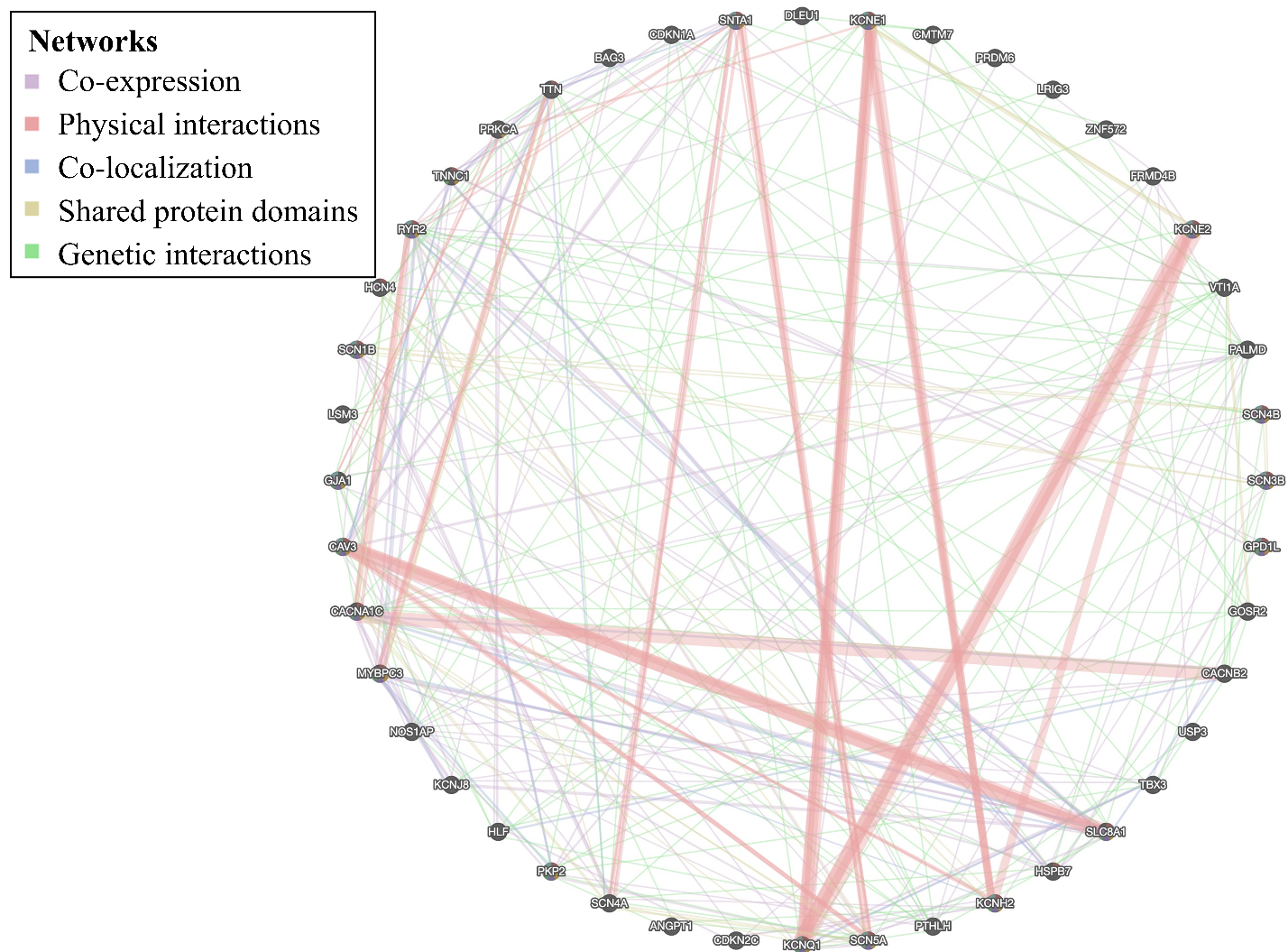


**Figure S3**

Network analysis was performed on random C4 (A) and C5 (B) genes. The colors correspond to the network types.


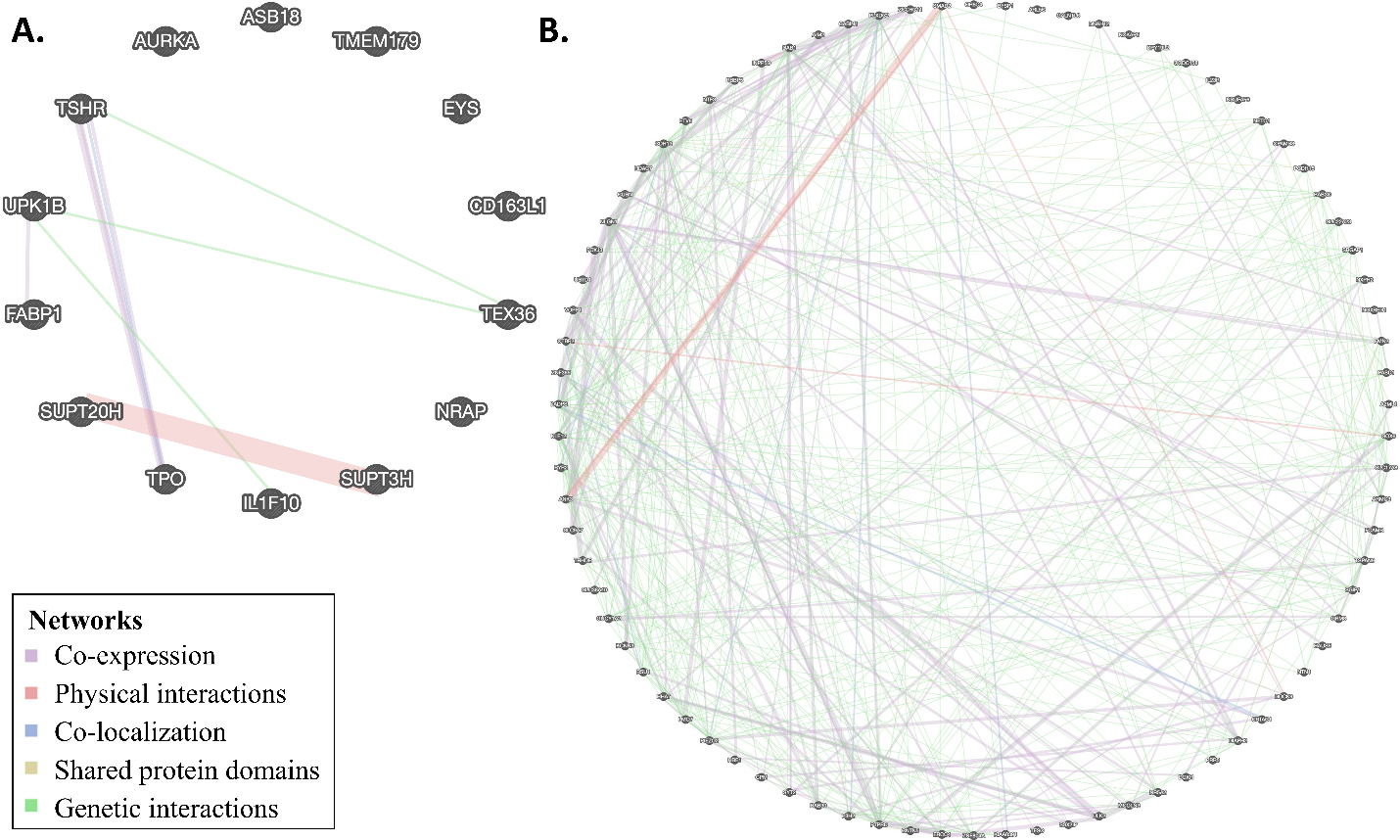

Supplement: Supplementary file 1 [file genes-12-00216-s001.zip › Johannsen et al. 2020 - Supp.docx]
